# Supplementary material for: Integration of mouse ovary morphogenesis with developmental dynamics of the oviduct, ovarian ligaments, and rete ovarii
Source: eLife. 2022 Sep 27;11:e81088. doi: 10.7554/eLife.81088 (PMC9621696; doi:10.7554/eLife.81088)
Supplement: Supplementary file 1. — (a) Color-coded table recapitulating ovarian and Müllerian duct phenotypes in the Pax2 and Pax8 allelic series (green with ✓, intact; yellow with ~, perturbed; red with X, absent). (b) Table recapitulating the DNA forward (middle) and reverse (right) primers used for genotyping the transgenic mouse lines used in the present study (right). (c) Table recapitulating the primary antibodies used in this study. Information in the table columns includes, from left to right protein recognized, host species, dilution, source, and product #. (d) Table recapitulating the secondary antibodies used in this study. Information in the table columns includes, from left to right protein recognized, dilution, source, and product #. [file elife-81088-supp1.docx]

**Supplementary Materials for**

**Integration of mouse ovary morphogenesis with developmental dynamics of the oviduct, ovarian ligaments, and rete ovarii.**

Jennifer McKey^1^, Dilara N. Anbarci^1^, Corey Bunce^1^, Alejandra E. Ontiveros^2^, Richard R. Behringer^2^, and Blanche Capel^1^*

*^1^Department of Cell Biology, Duke University Medical Center, Durham NC 27710*

*^2^Department of Genetics, University of Texas MD Anderson Cancer Center, Houston, TX, 77030*

| **Supplemental File 1a.** Ovarian and Müllerian duct phenotypes in *Pax2* and *Pax8* allelic series |
| --- |

|  |  | ***P2+/+ P8+/+*** | ***P2d/+ P8+/+*** | ***P2d/d P8+/+*** | ***P2+/+ P8d/+*** | ***P2+/+ P8d/d*** | ***P2d/+ P8d/+*** | ***P2d/d P8d/+*** | ***P2d/+ P8d/d*** |
| --- | --- | --- | --- | --- | --- | --- | --- | --- | --- |
| **Müllerian duct** | **Oviduct** | **✓** | **~** | **~** | **✓** | **~** | **~** | **X** | **X** |
|  | **Infundibulum** | **✓** | **✓** | **✓** | **✓** | **✓** | **✓** | **✓** | **✓** |
| **Rete ovarii** | **IOR** | **✓** | **✓** | **✓** | **X** | **X** | **~** | **X** | **X** |
|  | **CR** | **✓** | **✓** | **✓** | **~** | **X** | **~** | **~** | **X** |
|  | **EOR** | **✓** | **✓** | **X** | **✓** | **X** | **X** | **X** | **X** |
| **Ovary morphogenesis** | **Folding** | **✓** | **✓** | **✓** | **✓** | **✓** | **✓** | **✓** | **✓** |
|  | **Encapsulation** | **✓** | **✓** | **✓** | **✓** | **X** | **X** | **X** | **X** |
|  |  |  |  |  |  |  |  |  |  |
|  | **Legend** | **✓** | **~** | **X** |  |  |  |  |  |
|  |  | Intact | Perturbed | Absent |  |  |  |  |  |

| **Supplemental File 1b.** PCR Primers used in this study | | |
| --- | --- | --- |
|  |  |  |
| **Allele** | **Forward Primer** | **Reverse Primer** |
| *Pax2 wild-type* | AAAGTGAGGGAAGCGTAGAGAAG | ACCATAGACATTAGAGGTGCAGA |
| *Pax2del* | TGACTTTTGCAGTCCAGAGTCTCCC | ACCATAGACATTAGAGGTGCAGA |
| *Pax8 wild-type* | GAAAGTTCGAGGGAAGGGAGATC | CAGTTCTTTCAGTGGTCCCTCC |
| *Pax8del* | GAAAGTTCGAGGGAAGGGAGATC | GACCAAGTAGTCAATGGGGGCC |
| *Sf1:eGfp* | CACCATCTTCTTCAAGGACGAC | GTCACGAACTCCAGCAGGACC |
| *Sry* | GTGTCTCAAAGCCTGCTCTTC | CATGTACTGCTAGCAGCTATC |
| *Myogenin*  *(internal control)* | TTACGTCCATCGTGGACAGCAT | TGGGCTGGGTGTTAGTCTTAT |

#

#

| **Supplemental File 1c.** Primary antibodies used in this study | | | | |
| --- | --- | --- | --- | --- |
|  |  |  |  |  |
| **Primary Antibody** | **Host Species** | **Dilution** | **Source** | **Product #** |
| AMH/MIS | Goat | 1:500 | Santa Cruz Biotechnology | sc-6886 (discontinued) |
| aSMA  (Cy3 conjugated) | Mouse | 1:1000 | Sigma | C6198 |
| aSMA  (FITC conjugated) | Mouse | 1:500 | Sigma | F3777 |
| E-Cadherin | Rat | 1:500 | Zymed (Thermo Fischer) | 13-1900 |
| ENDOMUCIN | Rat | 1:250 | Santa Cruz Biotechnology | sc-65495 |
| FOXL2 | Goat | 1:250 | Novus Biologicals | NB100-1277 |
| GATA4 | Goat | 1:250 | Santa Cruz Biotechnology | sc-1237 (discontinued) |
| GFP | Chicken | 1:1000 | Abcam | ab13970 |
| HuC/D | Human | 1:10000 | Gift from V. Lennon  (Mayo Clinic) | N/A |
| KRT8 | Rat | 1:250 | DSHB | TROMA-I |
| PAX8 | Rabbit | 1:500 | Proteintech | 10336-1-AP |
| RUNX1 | Rabbit | 1:500 | Abcam | ab92336 |
| SOX9 | Rabbit | 1:1000 | Millipore | AB5535 |
| TNC | Rabbit | 1:250 | Gift from H. Erickson  (Duke University) | N/A |
| TUJ1 | Rabbit | 1:1000 | Abcam | ab18207 |

#

| **Supplemental File 1d.** Secondary antibodies used in this study | | | |
| --- | --- | --- | --- |
|  |  |  |  |
| **Secondary Antibody** | **Dilution** | **Source** | **Product #** |
| AF647 Donkey anti-**Rabbit** | 1:500 | Jackson ImmunoResearch | 711-605-152 |
| Cy3 Donkey anti-**Goat** | 1:500 | Jackson ImmunoResearch | 705-165-147 |
| Cy3 Donkey anti-**Chicken** | 1:500 | Jackson ImmunoResearch | 703-165-155 |
| AF488 Donkey anti-**Chicken** | 1:500 | Jackson ImmunoResearch | 703-545-155 |
| AF488 Donkey anti-**Human** | 1:500 | Jackson ImmunoResearch | 709-545-149 |
| AF488 Donkey anti-**Rat** | 1:500 | Life Technologies | A-21208 |
| Cy3 Donkey anti-**Rat** | 1:500 | Jackson ImmunoResearch | 712-165-150 |
|  |  |  |  |
| CF647-hydrazide probe  to label Elastin | 1:500 | Millipore Sigma | SCJ4600046 |

# 
